# Gut microbiota and metabolite variations in a migraine mouse model

**DOI:** 10.3389/fcimb.2023.1322059

**Published:** 2024-01-31

**Authors:** Dan Wang, Xu Liu, Suming Shi, Tongli Ren, Wuqing Wang

**Affiliations:** ^1^ Ear, Nose, and Throat (ENT) Institute and Department of Otorhinolaryngology, Eye & ENT Hospital, Fudan University, Shanghai, China; ^2^ National Health Council (NHC) Key Laboratory of Hearing Medicine, Fudan University, Shanghai, sChina

**Keywords:** migraine, gut microbiome, gut metabolome, 16S rRNA gene sequencing, microbiota-gut-brain axis

## Abstract

Migraine is a prevalent clinical disorder characterized by recurrent unilateral throbbing headache episodes accompanied by symptoms such as nausea, vomiting, photophobia, and phonophobia. Despite its common occurrence, the diagnosis, pathophysiology, and treatment of migraine remain controversial. Extensive research has implicated the gut microbiota in various central nervous system disorders, including anxiety disorders, depression, and Parkinson’s disease. Some studies have also suggested that migraine may stem from disruptions to neurohormones and metabolism. This study aimed to investigate the disparities in gut microbiota and metabolites between migraine mice model and normal mice to shed light on the underlying mechanisms and potential therapeutic approaches. Distinct differences in gut microbial composition were observed between the migraine mouse model and normal mouse, indicating a potential correlation between these variations and the pathogenesis of migraine. This study provides evidence of differences in gut microbiota composition and metabolites between a migraine mouse model and normal mice, which showed that Akkermansiaceae constituted the most abundant taxon in the sham injection mouse group, while Lachnospiraceae constituted the most prevalent group in the migraine mouse model group. The associations between the abundances of *Akkermansia muciniphila* and Lachnospiraceae bacteria and metabolites suggested their potential roles in the pathogenesis of migraine. The altered abundance of Lachnospiraceae observed in migraine-afflicted mice and its correlations with changes in metabolites suggest that it may affect the host’s health. Thus, probiotic therapy emerges as a possible treatment for migraine. Moreover, significant disparities in gut metabolites were observed between the migraine mouse model and normal mice. These alterations encompass multiple metabolic pathways, suggesting that metabolic disturbances may also contribute to the development of migraines.

## 1 Introduction

Migraine, a common neurological disorder, is characterized by recurrent unilateral throbbing headaches accompanied by symptoms such as nausea, vomiting, photophobia, and phonophobia (Schiffman et al., 2013). Despite its high prevalence, the diagnosis, pathophysiology, and treatment of migraine remain contentious. Migraine has been widely acknowledged as a distinct disorder associated with neurological disturbances. However, the precise mechanisms underlying its pathogenesis are not fully understood, and there is a lack of unified explanations for patient symptoms in both clinical studies and basic experiments.

The hypothesis that the activation of the trigeminovascular system can trigger the release of various neurotransmitters, such as calcitonin gene–related peptide and substance P leading to headaches, has gained wide acceptance (Vass et al., 2001; Vass et al., 2004). Another study has suggested that migraine results from neurohormonal and metabolic integrity loss (Dzugan and Dzugan, 2015). In recent years, the human gut microbiota has emerged as a focal point of research due to its role as the largest reservoir of human microbiota. It forms a symbiotic ecosystem with the host, influencing human health through metabolites, and interactions with the host’s immune system. Previously, the central nervous system was thought to influence the gut microbiota. However, with the rapid development of microbial sequencing technologies, it has become evident that the gut microbiota functions like an endocrine organ, producing various biologically active substances that enter the bloodstream and modulate distant organ functions (Tang et al., 2017). The gut microbiota exerts considerable influence on the central nervous system through multiple pathways. This realization has led to the concept of the “gut–brain axis” (Cryan et al., 2019). Abundant research has demonstrated the pivotal role of the gut microbiota in regulating various central nervous system disorders, such as anxiety disorders, depression, and Parkinson’s disease (Ma et al., 2019; Li et al., 2023). However, the mechanisms through which gut microbes influence the nervous system and their roles in disease pathogenesis remain unclear. Potential pathways through which this interaction occurs include the vagus nerve pathway, which connects the brain and the gut; the neuroendocrine pathway, especially the hypothalamic–pituitary–adrenal axis; metabolic pathways influenced by gut microbiota metabolites; and immune system pathways involving various immune factors (Décarie-Spain et al., 2023). Within the neuroendocrine pathway, the gut microbiota can affect the hypothalamic–pituitary–adrenal axis by modulating the secretion of 5-hydroxytryptamine(5-HT) from enteroendocrine cells into the peripheral bloodstream, thereby affecting the central nervous system (Kasarello et al., 2023). The gut microbiota produces specific metabolites, including amines (such as histamine, tyramine, putrescine, and cadaverine). For example, amines can be absorbed *in situ* and may affect the host’s behavior through unknown mechanisms, potentially contributing to central nervous system disorders (Gabastou et al., 1996). Alterations in the gut microbiota have been associated with various conditions, including metabolic disorders, obesity, hematological malignancies, neurological or behavioral disorders, and migraines. Metabolites produced by the gut microbiome have been shown to influence the gut–brain axis. The use of probiotics as dietary supplements may reduce the frequency and severity of migraine attacks, For instance, a study found that an IgG elimination diet combined with probiotics reduced migraine attack frequency in individuals with migraine and irritable bowel syndrome (Xie et al., 2019). Fecal microbiota transplantation represents a promising approach to treating migraine (Kappéter et al., 2023).

Therefore, this study established a mouse model of migraine to investigate the differences in gut microbiota and gut metabolites between migraine-afflicted mice and normal mice and to explore the mechanisms underlying the pathogenesis of migraine and potential therapeutic avenues.

## 2 Materials and methods

### 2.1 Experimental design and modeling

#### 2.1.1 Animals

C57BL/6J WT mice (C57BL/6-Htr1Dem1Smoc, aged 6–8 weeks) obtained from Nanmo Bio were used as experimental subjects and kept under a normal diet and regular 12 h light/dark cycles. All animal procedures were performed by the experimental protocol approved by the animal ethics committee of the medical school at Fudan University in China. This research involved a cohort of 40 mice, with 18 designated for the primary experimental protocols and 22 utilized for procedural training sessions.

#### 2.1.2 Experiment protocol

To augment the reliability of our experimental outcomes, the mice were divided into 3 groups, the gender ratio in each group of mice was 1:1, and we implemented two control groups: one subjected to no interventions (CON: n = 6) and another administered intraperitoneal injections of physiological saline solution(NS: n = 6), the migraine model group (CM: n = 6). The CM group was intraperitoneally injected with nitroglycerin (10 mg/kg) every other day for nine consecutive days (Demartini et al., 2019). The NS group received intraperitoneal injections of an equivalent volume of 0.9% saline. The CON group (CON) did not receive any intervention.

#### 2.1.3 Pain threshold detection

Using von Frey mechanical stimulation filaments (Stoelting), changes in mechanical stimulation sensitivity thresholds around the eyes and hind paws of the mice were measured on days 1, 3, 5, 7, and 9 of modeling and days 1, 3, 5, 7, and 9 after modeling. A series of von Frey filaments with incrementally increasing stimulus forces (0.07, 0.16, 0.40, 0.60, 1.00, 1.40, and 2.00 g) were used. To acclimate themselves to the testing environment, the mice were placed in it for 1 h/day for two to three consecutive days before testing. The temperature and humidity of the experimental environment were kept stable. The sensitivity thresholds were recorded.

### 2.2 16S rRNA gene sequencing of the gut microbiome

Total genome DNA from samples was extracted using the cetyltrimethylammonium bromide method. DNA concentration and purity was monitored on 1% agarose gels. According to the concentration, DNA wasdiluted to 1ng/L using sterile water.16S rRNA genes of distinct regions (16S V4/16S V3/16S V3-V4/16SV4-V5) were amplified used the specific primer (16SV4: 515F- 806R), with the barcode. All PCR reactions were carried out with 15 L of Phusion® High -Fidelity PCR Master Mix (New England Biolabs); mixture PCR products was purified with Qiagen Gel Extraction Kit (Qiagen, Germany). Sequencing libraries were generated usingTruSeq® DNA PCR-Free Sample Preparation Kit(Illumina, USA) following manufacturer’s recommendations and index codes were added.The library quality was assessed on the Qubit@ 2.0 Fluorometer (Thermo Scientific) and Agilent Bioanalyzer 2100 system. At last, the library was sequenced on an Illumina NovaSeq platform and 250 bp paired-end reads were generated.

In order to study phylogenetic relationship of different OTUs, and dominant species in different samples (groups), multiple sequence alignment were conducted using the MAFFT (v7.490, https://mafft.cbrc.jp/alignment/software/) (Caporaso et al., 2010). OTUs abundance information were normalized using a standard of sequence number corresponding to the sample with the least sequences. Subsequent analysis of alpha diversity and beta diversity were all performed basing on this output normalized data. Alpha diversity is applied in analyzing complexity of species diversity for a sample through 6 indices, including Observed-species, Chao1, Shannon, Simpson, ACE, Good-coverage. All this indices in our samples were calculated with QIIME and displayed with R software (Version 4.1.2). Beta diversity analysis was used to evaluate differences of samples in species complexity, Beta diversity on both weighted and unweighted unifrac were calculated by QIIME software. Cluster analysis was preceded by principal component analysis (PCA), which was applied to reduce the dimension of the original variables using the stats package and ggplot2 package in R software. Principal Coordinate Analysis (PCoA) was performed to get principal coordinates and visualize from complex, multidimensional data. A distance matrix of weighted or unweighted unifrac among samples obtained before was transformed to a new set of orthogonal axes, by which the maximum variation factor is demonstrated by first principal coordinate, and the second maximum one by the second principal coordinate, and so on. PCoA analysis was displayed by stats package and ggplot2 package in R software. Unweighted Pair-group Method with Arithmetic Means (UPGMA) Clustering was performed as a type of hierarchical clustering method to interpret the distance matrix using average linkage and was conducted by QIIME software.

### 2.3 Gut metabolomics analysis

The original data file acquisited by LC-MS was converted into mzML format by ProteoWizard software. Peak extraction, peak alignment and retention time correction were respectively performed by XCMS program. The “SVR” method was used to correct the peak area. The peaks with detetion rate lower than 50% in each group of samples were discarded. After that, metabolic identification information was obtained by searching the laboratory’s self-built database, integrated public database, AI database and metDNA.

Unsupervised PCA (principal component analysis) was performed by statistics function prcomp within R (www.r-project.org). The data was unit variance scaled before unsupervised PCA.The HCA (hierarchical cluster analysis) results of samples and metabolites were presented as heatmaps with dendrograms, while pearson correlation coefficients (PCC) between samples were caculated by the cor function in R and presented as only heatmaps. Both HCA and PCC were carried out by R package ComplexHeatmap. For HCA, normalized signal intensities of metabolites (unit variance scaling) are visualized as a color spectrum.

For two-group analysis, differential metabolites were determined by VIP (VIP > 1) and P-value (P-value < 0.05, Student’s t test). For multi-group analysis, differential metabolites were determined by VIP (VIP > 1) and P-value (P-value < 0.05, ANOVA). VIP values were extracted from OPLS-DA result, which also contain score plots and permutation plots, was generated using R package MetaboAnalystR. The data was log transform (log22) and mean centering before OPLS-DA. In order to avoid overfitting, a permutation test (200 permutations) was performed.

Identified metabolites were annotated using KEGG Compound database (http://www.kegg.jp/kegg/compound/), annotated metabolites were then mapped to KEGG Pathway database (http://www.kegg.jp/kegg/pathway.html). Significantly enriched pathways are identified with a hypergeometric test’s P-value for a given list of metabolites.

### 2.4 Statistical analysis

The Wilcoxon signed-rank test or Student’s t-test was used for comparisons between multiple groups. Spearman’s correlation coefficients were used to determine the correlations between gut microbiota and metabolites. P-values of <0.05 were considered statistically significant. Figures and charts were created using R software (Version 4.1.2).

## 3 Results

### 3.1 Pain threshold

**Figure 1:**
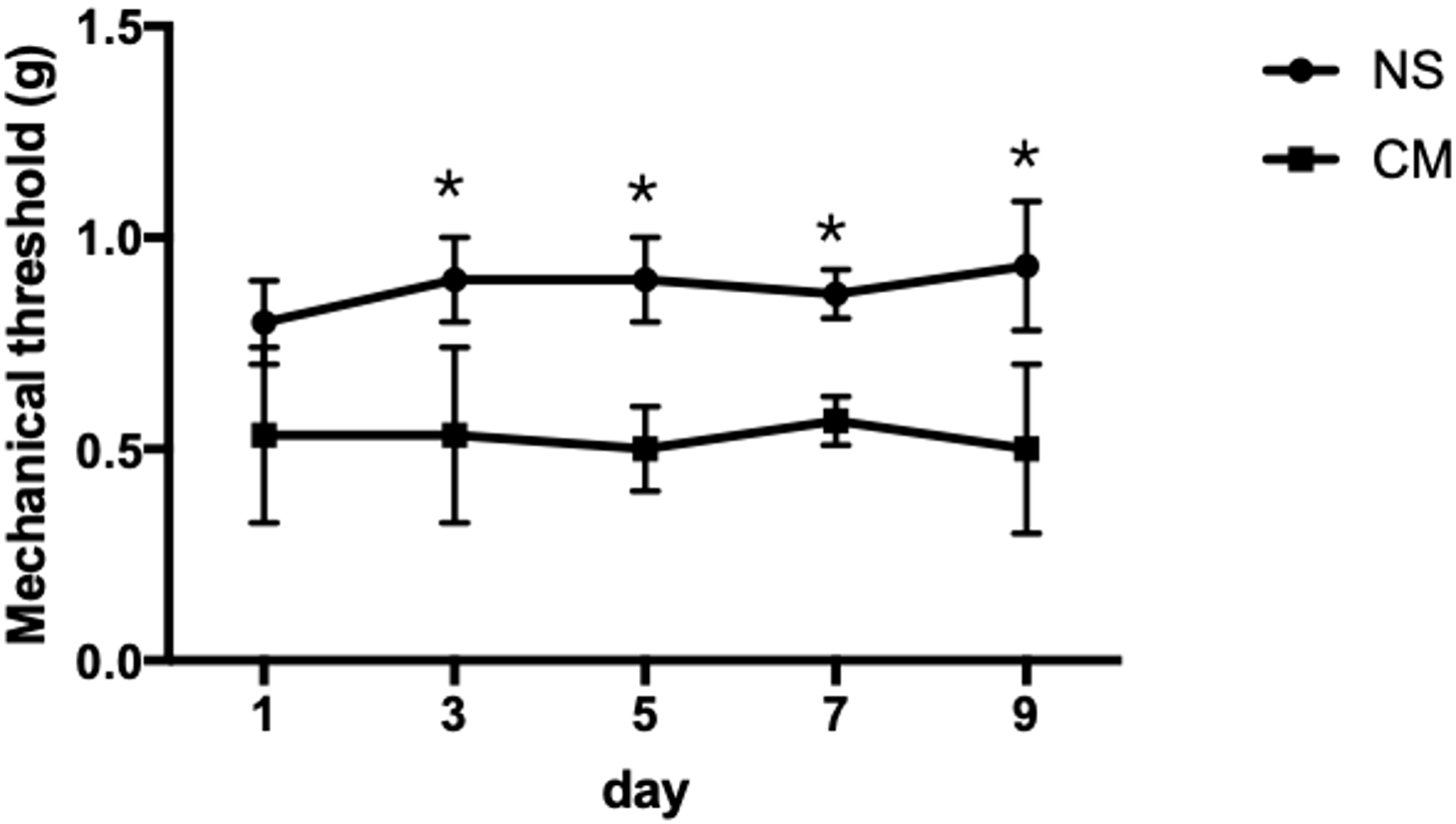
This line chart demonstrates the mechanical stimulus sensitivity thresholds of the NS and CM groups assessed on days 1, 3, 5, 7, and 9 after modeling. The pain threshold of the CM group was notably lower than that of the NS group. *P<0.05, the differences are statistically significant.

The pain thresholds of mice in the CM group remained consistently lower than those in the NS group (p < 0.05; **Figure 1**). This confirmed the successful establishment of the migraine model, and no adverse or toxic effects were observed in the model.

### 3.2 Mouse gut microbiota

#### 3.2.1 Microbial diversity analysis

**Figure 2:**
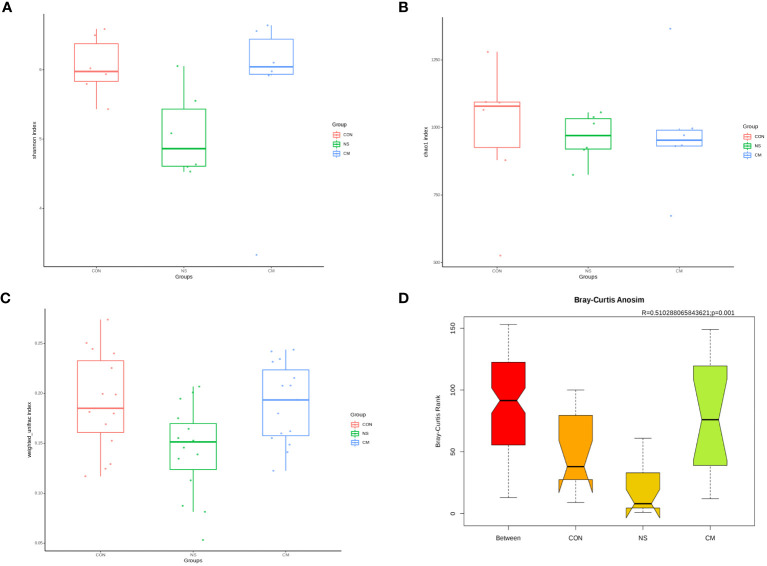
Alpha diversity of gut bacteria: Box plots representing **(A)** community diversity and **(B)** richness were used to estimate the alpha diversity in the CM (blue), CON (red), and NS (green) groups. The boxes show the medians, interquartile ranges, and minimum and maximum values. Beta diversity of gut microbiota: **(C)** Box plot based on OTUs’ unweighted UniFrac beta diversity. **(D)** ANOSIM (r= 0.51, p = 0.001) revealed significant differences between the NS and CM groups.

The Shannon index (**Figure 2A**) and the richness estimator Chao1 (**Figure 2B**) revealed no significant differences in gut microbiota diversity between the three groups.However, the unweighted_unifrac WilcoxTest analysis (**Figure 2C**) revealed significant differences in beta diversity between the NS and CM groups (p = 0.01). Moreover, ANOSIM (**Figure 2D**) revealed significant differences between the three groups (r = 0.51, p = 0.001).

#### 3.2.2 Species difference and contribution analysis

**Figure 3:**
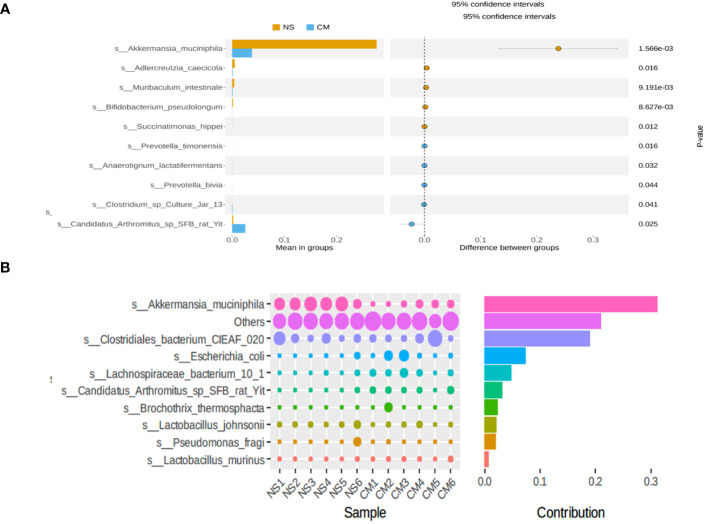
**(A)** Statistical analysis of gut microbiota in the CM and NS groups based on Student’s t-test. The abundance of *Akkermansia muciniphila* was significantly higher in the NS group than in the CM group. **(B)** SIMPER analysis showing the top 10 species contributing to the differences between the NS and CM groups. *Akkermansia muciniphila* had the greatest contribution.

Fecal 16S rRNA sequencing and t-tests revealed significant intergroup differences in species based on OTUs. Notably, the abundance of *Akkermansia muciniphila* was markedly higher in the NS group than in the CM group (**Figure 3A**). *Akkermansia muciniphila* exhibited the greatest dissimilarity between the NS and CM groups, with a substantially greater abundance in the former than in the latter (**Figure 3B**).

#### 3.2.3 Comparison of population abundances

**Figure 4:**
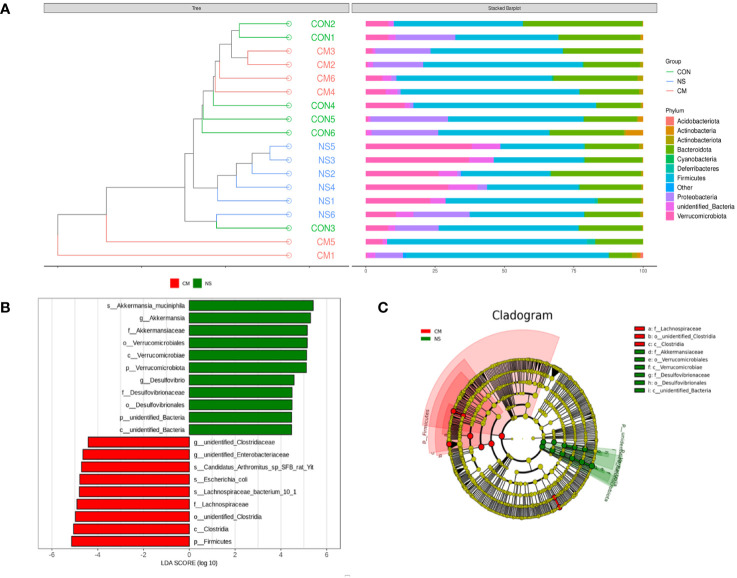
**(A)** The UPGMA clustering analysis indicates the differences between the three groups at the phylum level. he brightness of each point corresponds to the magnitude of its LDA effect size **(B)**. The LDA scores are combined with the effect size measurements in the CM and NS groups. Abundant taxonomic groups in the NS (green) and CM (red) groups are displayed along with the LDA scores. Only taxonomic groups with LDA scores exceeding the threshold of 4.0 are displayed **(B)**. **(C)** Cladogram representing the gut microbiota in the CM and NS groups constructed using 16S rRNA sequencing. Abundant taxonomic groups are indicated for both the NS (green) and CM (red) groups.

The results of the UPGMA clustering analysis (**Figure 4A**) indicated differential relative abundances between the groups at the phylum level. The linear discriminant analysis (LDA) value distribution histogram obtained from the LEfSe analysis (**Figure 4B**) also revealed microbes that were differentially abundant in the NS and CM groups. The NS group was characterized by a high abundance of *Akkermansia muciniphila*, while the CM group was characterized by a high abundance of unidentified Clostridiaceae. A phylogenetic tree (**Figure 4C**) showed that Akkermansiaceae constituted the most abundant taxon in the NS group, while Lachnospiraceae constituted the most prevalent group in the CM group.

### 3.3 Mouse gut metabolomics

#### 3.3.1 Metabolite PCA

**Figure 5:**
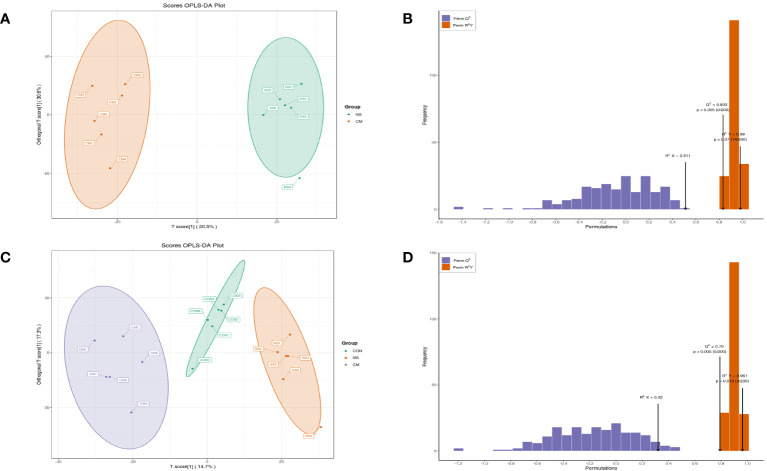
OPLS-DA analysis was performed to show the metabolite profile (NS: n = 6, CON: n = 6, CM: n = 6). The OPLS-DA score and model validation plots showed clear discrimination between the NS and CM groups, with R2X = 0.511, R2Y = 0.980, Q2 = 0.833, and p < 0.05 **(A, B)**. They also showed clear discrimination between the NS, CM, and CON groups, with R2X = 0.320, R2Y = 0.961, Q2 = 0.790, and p < 0.05 **(C, D)**.

A PCA plot of metabolites was conducted for the NS and CM groups (**Figure 5A**) and for the VM, NS, and CON groups (**Figure 5C**). The results showed distinct intergroup differences in the gut metabolomes of the mice. The orthogonal partial least squares discriminant analysis (OPLS-DA) scores indicated clear discrimination between the NS, CM, and CON groups (**Figures 5A, C**). The goodness of fit values and predictive ability values (NS vs. CM: R2X = 0.511, R2Y = 0.980, Q2 = 0.833, p < 0.05; CON vs. CM and CON: R2X = 0.320, R2Y = 0.961, Q2 = 0.790, p < 0.05) showed that the OPLS-DA model exhibited satisfactory fitting and great predictive ability (**Figures 5B, D**).

#### 3.3.2 Differential metabolites

**Figure 6:**
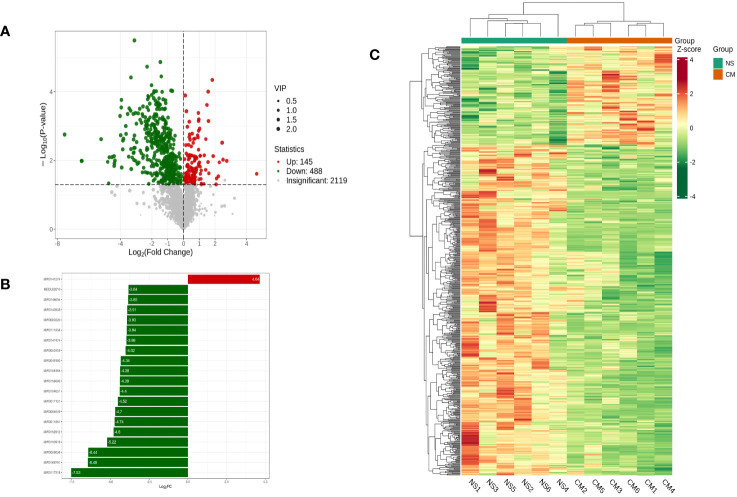
Volcano plot showing differential metabolites between the NS and CM groups **(A)**. Linear discriminant analysis (LDA) effect size (LEfSe) results showed the top 20 differential metabolites between the NS and CM groups **(B)**. Hierarchical cluster analysis of differential metabolites between the NS and CMgroups **(C)**. Each column represents a sample, and each row stands for a metabolite. Each column represents a sample, and each row represents a metabolite. The differential metabolites were determined using the following criteria: VIP ≥ 1.0, p < 0.05, and fold change ≥ 2 or ≤ 0.5.

Based on the OPLS-DA results, we used variable importance in projection (VIP) as a threshold to further screen for metabolite differences between the groups, considering metabolites with a VIP value of ≥1.0, a p-value of <0.05, and a fold change value of ≥2 or ≤0.5 to be differentially regulated. A total of 633 differential metabolites were detected in the NS and CM groups. Of those, 145 showed significantly higher levels in the CM group than in the NS group, while 488 exhibited significantly lower levels (**Figure 6**).

#### 3.3.3 Metabolic pathway analysis

**Figure 7:**
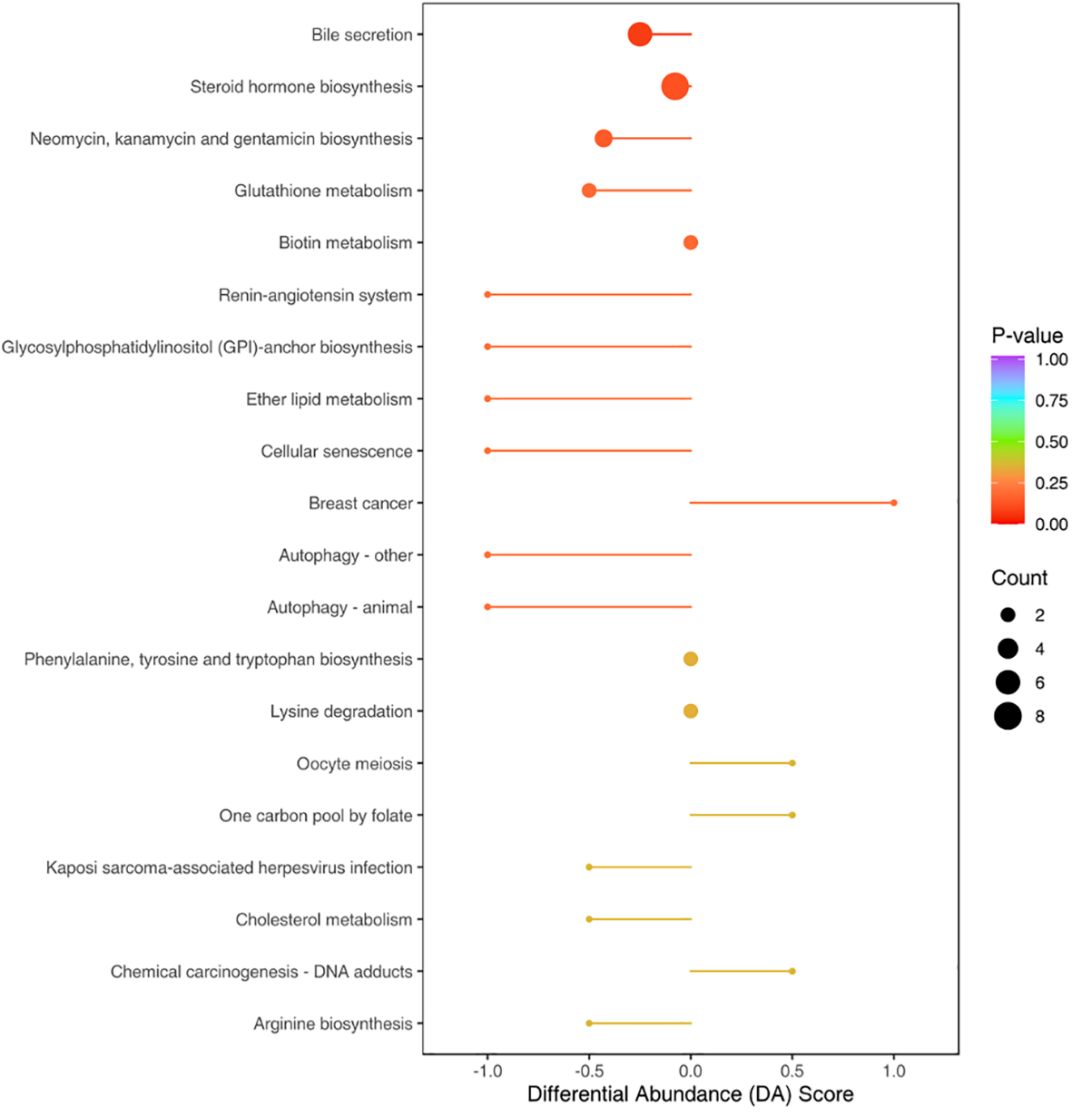
Top 20 pathways with the most pronounced differences in gut metabolites between the NS and CM groups. The size of each origin corresponds to the number of differential metabolites, while the colors indicate different levels of statistical significance. The differential metabolites were primarily associated with pathways such as bile secretion, steroid hormone biosynthesis, biosynthesis of neomycin and kanamycin, glutathione metabolism, and biotin metabolism.

To identify the involved metabolic pathways, the differential metabolites were examined against the KEGG database. The results revealed the involvement of various metabolic pathways (**Figure 7**). Some of the main pathways were bile secretion, steroid hormone biosynthesis, biosynthesis of neomycin and kanamycin, glutathione metabolism, and biotin metabolism.

### 3.4 Relationship between differential microbes and differential metabolites

**Figure 8:**
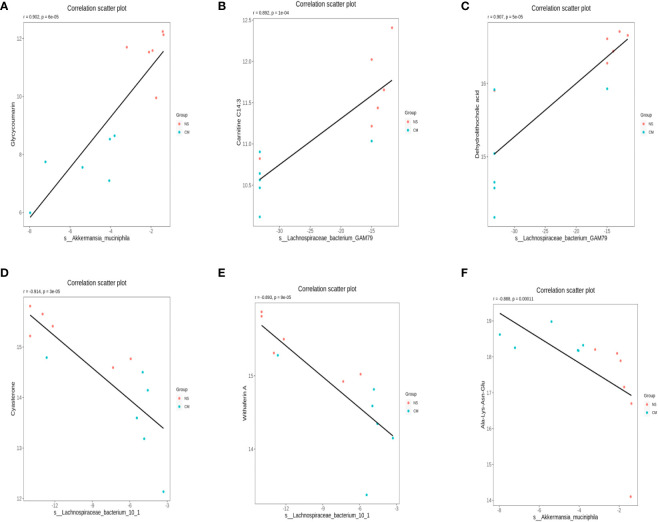
Scatterplots illustrating significant correlations between the altered relative abundances of gut bacterial genera and the LC-MS spectral intensities of differential metabolites. **(A)**: the correlations between the abundances of S-Akkermansia muciniphila and glycycoumarin is positive, **(B)** and **(C)**: the correlations between the abundances of Lachnospiraceae bacterium GAM79S and dehydrolithocholic acid and carnitine C14:3 is positive. **(D)** and **(E)**: the correlations between the abundances of Lachnospiraceae bacterium 10-1 and withaferin A and cysteine is negative, **(F)** the correlations between the abundances of S-Akkermansia muciniphila and Ala-Lys-Asn-Glu is negative.

We calculated Spearman’s correlation coefficients to determine the functional correlations between variations in gut microbiota and metabolite profiles in the groups, with r ≥ 0.8 and p < 0.05 considered to indicate significant correlations. The results (**Figure 8**) indicated the following correlations between the abundances of specific microbes and metabolites: *Akkermansia muciniphila*–glycycoumarin (positive), Lachnospiraceae bacterium GAM79S–dehydrolithocholic acid and carnitine C14:3 (positive), *Akkermansia muciniphila*–Ala-Lys-Asn-Glu (negative), and S-Lachnospiraceae bacterium 10-1–withaferin A and cysteine (negative).

## 4 Conclusion

This study provides evidence of differences in gut microbiota composition and metabolites between a migraine mouse model and normal mice, which showed that Akkermansiaceae constituted the most abundant taxon in the NS group, while Lachnospiraceae constituted the most prevalent group in the CM group. The associations between the abundances of *Akkermansia muciniphila* and Lachnospiraceae bacteria and metabolites suggested their potential roles in the pathogenesis of migraine. The altered abundance of Lachnospiraceae observed in migraine-afflicted mice and its correlations with changes in metabolites suggest that it may affect the host’s health. Overall, this study provides insights into the potential roles of the gut microbiota and metabolites in migraines, indicating potential research avenues and therapeutic strategies.

## 5 Discussion

In this study, we observed a decrease in the abundance of *Akkermansia muciniphila* in the guts of migraine-afflicted mice. A combined analysis of differential metabolites and differential bacteria revealed a positive correlation between the abundance of *Akkermansia muciniphila* and glycycoumarin. Glycycoumarin, a representative coumarin compound, exhibits significant pharmacological activities, including antitumor, hepatoprotective, antispasmodic, antibacterial, and antiviral effects (Tang et al., 2023). We also found a negative correlation between the abundance of *Akkermansia muciniphila* and the molecular substances named Ala-Lys-Asn-Glu. The biological effects of this peptide segment remain unexplored. However, they may be related to amino acid metabolism.

*Akkermansia muciniphila*, a Gram-negative anaerobic mucinophilic bacterium, is a unique representative of the Verrucomicrobia phylum. It thrives in the mucus layer of the intestinal epithelium by utilizing mucus as a source of carbon and nitrogen. It has been associated with improving host metabolic functions and immune responses, which makes it a promising candidate for probiotic therapy (Derrien et al., 2004; Zhang et al., 2019; Zhou and Zhang, 2019). A decrease in the abundance of *Akkermansia muciniphila* in the guts of migraine-afflicted mice and its correlation with specific metabolites, such as glycycoumarin, indicate potential protective action against migraine (Cani et al., 2022). Recent studies have indicated that *Akkermansia* in the gut can regulate serum 5-HT concentrations and influence central 5-HT levels through the gut–brain axis, suggesting a novel therapeutic strategy for treating 5-HT-related disorders (Yaghoubfar et al., 2020; Yaghoubfar et al., 2021; Sun et al., 2023). Certainly, some studies indicate exercise might reduce migraine frequency, however, the optimal exercise and intensity lack consensus. Moreover, evidence suggests vigorous exercise might provoke migraines (Amin et al., 2018). Nevertheless, contradictory findings show reduced Akkermansia levels in the gut microbiota of elite athletes (Hintikka et al., 2022).Perhaps high-intensity exercise could lead to a reduction in Akkermansia abundance, which might have a detrimental effect on migraine control.

In our study, the abundance of Lachnospiraceae in the guts of migraine-afflicted mice was greater than in those of normal mice. A combined analysis of differential metabolites and differential bacteria revealed a positive correlation between carnitine C14:3 and the abundance of Lachnospiraceae bacterium GAM79S. Carnitine plays a crucial role in energy metabolism. However, many of its functions remain unclear (Borum, 1983). We also found a negative correlation between cyasterone and S-Lachnospiraceae bacterium 10-1. Cyasterone can promote mesenchymal stem cell migration and osteogenesis, accelerating bone fracture healing (Zhu et al., 2021). Furthermore, we found a positive correlation between dehydrolithocholic acid and Lachnospiraceae bacterium GAM79S. We also found a negative correlation between withaferin A and S-Lachnospiraceae bacterium 10-1. Withaferin A is a C28 steroidal lactone derived from the plant *Withania somnifera*, commonly known as ashwagandha. It has attracted considerable attention due to its anticancer properties, which have been observed in various cancer cell lines from different sources, as well as its anti-inflammatory, metabolic, and proapoptotic activities (Atteeq, 2022). Our findings suggest that changes in the abundance of Lachnospiraceae can lead to alterations in gut metabolites and that different bacteria have different effects on metabolites, which, in turn, have different effects on the host’s health. Based on our findings, we can speculate that S-Lachnospiraceae bacterium 10-1 has adverse effects on the host’s health, while Lachnospiraceae bacterium GAM79S plays a beneficial role.

Different taxonomic groups within the Lachnospiraceae family have been associated with various diseases, and their impacts on host physiology often vary (Vacca et al., 2020). According to one analysis, the diversity of Lachnospiraceae may affect the ability of isolates to influence the host’s health (Sorbara et al., 2020). Schizophrenia is a debilitating psychiatric disorder with undefined underlying molecular mechanisms. The gut microbiota can regulate brain function and behavior through the microbiota–gut–brain axis. A previous study found a lower gut microbiota alpha diversity index and significantly greater gut microbial dysbiosis in both medicated and medication-naive SCZ patients than in healthy controls. The same study reported that several unique bacterial taxa, such as Veillonellaceae and Lachnospiraceae, were associated with the severity of SCZ (Zheng et al., 2019). *Fusimonas intestini*, a commensal species of the Lachnospiraceae family, highly colonizes obese and hyperglycemic humans and mice, producing long-chain fatty acids, such as elaidic acid, thus promoting diet-induced obesity (Takeuchi et al., 2023). However, another study reported that the abundances of unclassified Mogibacteriaceae, *Lachnospira*, and *Slackia* exhibited significant negative correlations with dehydrolithocholic acid (Liu et al., 2022).

In this study, we found significant differences in metabolites between the migraine mouse model (CM group) and the normal mice (NS group). The pathways most closely associated with these differential metabolites included bile secretion, steroid hormone biosynthesis, biosynthesis of neomycin and kanamycin, glutathione metabolism, and biotin metabolism. Among them, bile secretion and steroid hormone biosynthesis were the most severely affected, suggesting that abnormalities in bile and steroid metabolism may be associated with the pathogenesis of migraine. Regrettably, we did not establish a baseline comparison in animals before modeling. Our analysis of the gut microbiota in mice occurred post-modeling. Furthermore, our study lacked testing at various intervals, precluding precise determination of the timeline for changes in the gut microbiota of the animal model. Overall, this study provides insights into the potential roles of the gut microbiota and metabolites in migraines, indicating potential research avenues and therapeutic strategies, For instance, a study found that an IgG elimination diet combined with probiotics reduced migraine attack frequency in individuals with migraine and irritable bowel syndrome (Xie et al., 2019). However, given the current ambiguity surrounding the relationship between migraines and changes in gut microbiota, additional investigations are warranted. Consequently, evaluating its efficacy in mitigating migraines remains a complex task at present.Firstly, we need to ascertain the probiotics’ effectiveness timeline in the model, conducting pain threshold assessments at different intervals will provide insights into the onset of probiotic efficacy based on the pain threshold variations observed in mice. Secondly, For human subjects, prospective cohort studies are imperative to scrutinize the gut microbiota at different disease stages within the same patient, allowing inferences about the timing of microbiota alterations. Concerning the efficacy of probiotics, evaluating migraine symptom severity in patients post-probiotic treatment will facilitate discernment of the drug’s onset of action.

The study’s limitations include its small sample size and a lack of clinical validation. Further studies with larger samples and clinical investigations are necessary to confirm our findings. Furthermore, this study lacks an analysis of changes in pain thresholds in mice models post-fecal transplantation. This aspect will be addressed in our subsequent research endeavors. Moreover, this study focused on the acute stage of migraine. Future studies should conduct longitudinal observations and investigate the potential dynamic changes in the gut microbiota occurring during the development and treatment of migraine. Blood samples should be concurrently analyzed to validate the functional effects of differential metabolites on remote organs.

## Data availability statement

The accession number of our sequencing data is PRJNA1061318 in the SRA database and has been released successfully.

## Ethics statement

The animal study was approved by The ethical committee of the EENT Hospital. The study was conducted in accordance with the local legislation and institutional requirements.

## Author contributions

DW: Formal analysis, Methodology, Validation, Writing – review & editing. XL: Data curation, Formal analysis, Writing – original draft, Writing – review & editing. SS: Data curation, Writing – review & editing, Investigation. TR: Data curation, Investigation, Writing – review & editing. WW: Funding acquisition, Project administration, Supervision, Validation, Writing – review & editing.
